# Preserving the Membranous Labyrinth With a Piezoelectric Drill? Exploring Potential for Semicircular Canal Surgery

**DOI:** 10.1097/MAO.0000000000004990

**Published:** 2026-07-06

**Authors:** Joost Johannes Antonius Stultiens, Angelo Immordino, Nils Guinand, Raymond van de Berg

**Affiliations:** a Department of Otorhinolaryngology-Head and Neck Surgery, School for Mental Health and Neuroscience, Faculty of Health Medicine and Life Sciences, Maastricht University Medical Center, Maastricht, The Netherlands; b Department of Clinical Neurosciences, Division of Otorhinolaryngology-Head and Neck Surgery, Geneva University Hospitals, University of Geneva, Geneva, Switzerland

**Keywords:** Hearing, Inner Ear, Piezosurgery, Semicircular Canals, Vestibular Function, Vestibular Implant

## Abstract

**Hypothesis:**

A piezoelectric drill can fenestrate semicircular canals while preserving the membranous labyrinth.

**Background:**

Semicircular canal surgery carries a high risk of impairing auditory and vestibular function, limiting potential benefit to a small subset of patients. A piezoelectric drill might selectively disintegrate bone while sparing the membranous labyrinth and its endolymphatic compartment. Such selectivity could be particularly advantageous for patients who may benefit from vestibular implantation or semicircular canal plugging but have functional hearing and/or vestibular function.

**Methods:**

Formalin-fixed human temporal bones were included after skeletonizing (‘bluelining’) the semicircular canals. A piezoelectric drill with an oval-shaped osteoplasty insert was then used to drill 2 fenestrations in each semicircular canal. These locations were cleaned, and the integrity of the membranous labyrinth was assessed at each fenestration site with a surgical microscope.

**Results:**

In total, 60 fenestrations were made in 30 semicircular canals from 10 temporal bones. The membranous labyrinth was intact at all these fenestration sites.

**Conclusion:**

Piezoelectric surgery appears feasible for consistently fenestrating the semicircular canals while preserving the membranous labyrinth. It may enable safer semicircular canal procedures, such as vestibular implantation, in patients with residual inner ear function.

## INTRODUCTION

The use of surgical drills has become a key facet of temporal bone surgery. Although chisels can still be useful for particular applications, the introduction of rotating drills has enabled both efficient and precise removal of dense bone. Cutting burrs featuring sharp blades enable efficient local drilling, while diamond-coated burrs—without blades—provide a more localized effect to rather abrade the bone, eliminating the risk of blade-related injury to soft tissue. Nevertheless, the rotational force itself can still cause mechanical or thermal damage to adjacent soft tissue.

A particularly vulnerable structure is the membranous labyrinth. This structure of the inner ear separates the endolymph from the surrounding perilymph.^1^ When the bony labyrinth is opened, as in semicircular canal plugging or vestibular implantation, the membrane may be ruptured by the rotating drill. For example, in the intralabyrinthine semicircular canal approach for vestibular implantation, a fenestration is made in each semicircular canal to allow advancement of an electrode to its ampulla. There, the electrode serves to stimulate the adjacent neural tissue, to facilitate the restoration of vestibular function. Surgically fenestrating and then plugging a semicircular canal may be indicated for certain patients with disabling superior canal dehiscence syndrome or benign paroxysmal positional vertigo. While inner ear function may be present in candidates for these procedures, hearing and vestibular function are at risk.^2–4^ Deterioration might occur due to rupture of this membrane, as it could cause leakage of endolymph, mixing of endolymph and perilymph, as well as mechanical stress on the sensory structures. Therefore, methods to spare the membranous labyrinth could play a crucial role in reducing the risk of the most debilitating adverse effect associated with inner ear surgery. By minimizing these risks, a broader range of patients could benefit from these procedures.

The risk of rupture when opening the semicircular canals can be minimized by meticulously skeletonizing (‘bluelining’) and then using a microhook to remove the very thin remaining bone or endosteum.^5^ However, to use this method, the remaining bone must be drilled very thin, which increases the risk of accidentally opening the canal. In addition, the hook risks damaging the membrane. A recently investigated hand-guided robotic drill that relies on force and torque sensing parameters to automate cessation,^6^ provided promising results for drilling through bone while sparing the underlying membranous labyrinth.^7^ However, this drill is still under development and not yet clinically available.

Piezoelectric bone surgery is clinically employed for various osseous procedures. It uses ultrasonic vibrations to selectively dissect bone. It utilizes the inverse piezoelectric effect, converting electrical energy into mechanical energy. With a relatively low frequency (ca. 24 to 36 kHz) and micrometric amplitude (around 10 to 250 μm), mineralized tissue can be selectively fractured and fragmented. Soft tissue dissection occurs at higher amplitudes and frequencies. As such, soft tissue vibrates but is not disrupted.^8^ Consequently, with the right set-up, the (blunt) tool could theoretically be used to cut through the bony semicircular canal while sparing the membranous labyrinth.

A cadaveric human temporal bone trial could serve as the first step to investigate the feasibility of this approach for patients. Therefore, the current study was designed to determine whether piezoelectric surgery can be used to fenestrate the semicircular canals without rupturing the membranous labyrinth.

## MATERIALS AND METHODS

### Materials

Formalin-fixed cadaveric human temporal bones were used. These were prepared for the study with a rotating drill as regularly used in otology practice (Visao, Medtronic, Dublin, Ireland). A mastoidectomy was performed and the bony semicircular canals were exposed. This rotating drill was also employed to skeletonize the semicircular canals to uncover their course (‘bluelining’), using a fine diamond burr. Edges around and lateral to the blueline were removed. The succeeding study procedures were performed using a piezoelectric instrument (Piezosurgery plus, Mectron S.p.A., Carasco, Italy), with an oval-shaped osteoplasty insert (MP3-a30; width: 2.4 mm, thickness: 0.8 mm). The device was set to low power and low irrigation (Piezosurgery plus settings: power on 2, irrigation on 1). The insert and settings were determined based on a pilot trial.

### Experimental Procedures

The piezoelectric drill was used to fenestrate the semicircular canals. The insert was pushed forward over the blueline repeatedly until a fenestration occurred (see Discussion for best practices). Two separate fenestrating spots were chosen for each semicircular canal, without any fixed order. The fenestration was made over a length of at least 1 mm to adequately visualize the contents of the semicircular canal. A surgical microscope (OPMI Pentero, Carl Zeiss Surgical GmbH, Oberkochen, Germany) was used to inspect the fenestration and determine whether the membranous labyrinth was intact (2 authors, J.S. and R.v.d.B., in consensus). If necessary for visualization, water was splashed on the fenestration to rinse away bone fragments, or the relatively blunt oval-shaped drill insert was used to scrape away remaining fragments.

### Analysis

Descriptive statistics were used. The number and the proportion of fenestrations that could be made without damaging the membranous labyrinth were determined as the main outcome measures of this study.

### Ethics

This study was made possible thanks to anatomical donors. The Department of Anatomy and Embryology of the concerning university stores handwritten and signed codicils from these donors, in compliance with the Dutch Corpse Disposal Act (“Wet op de lijkbezorging”, 1991). The procedures undertaken in this investigation complied with Dutch legislation and ethical standards.

## RESULTS

**Figure 1:**
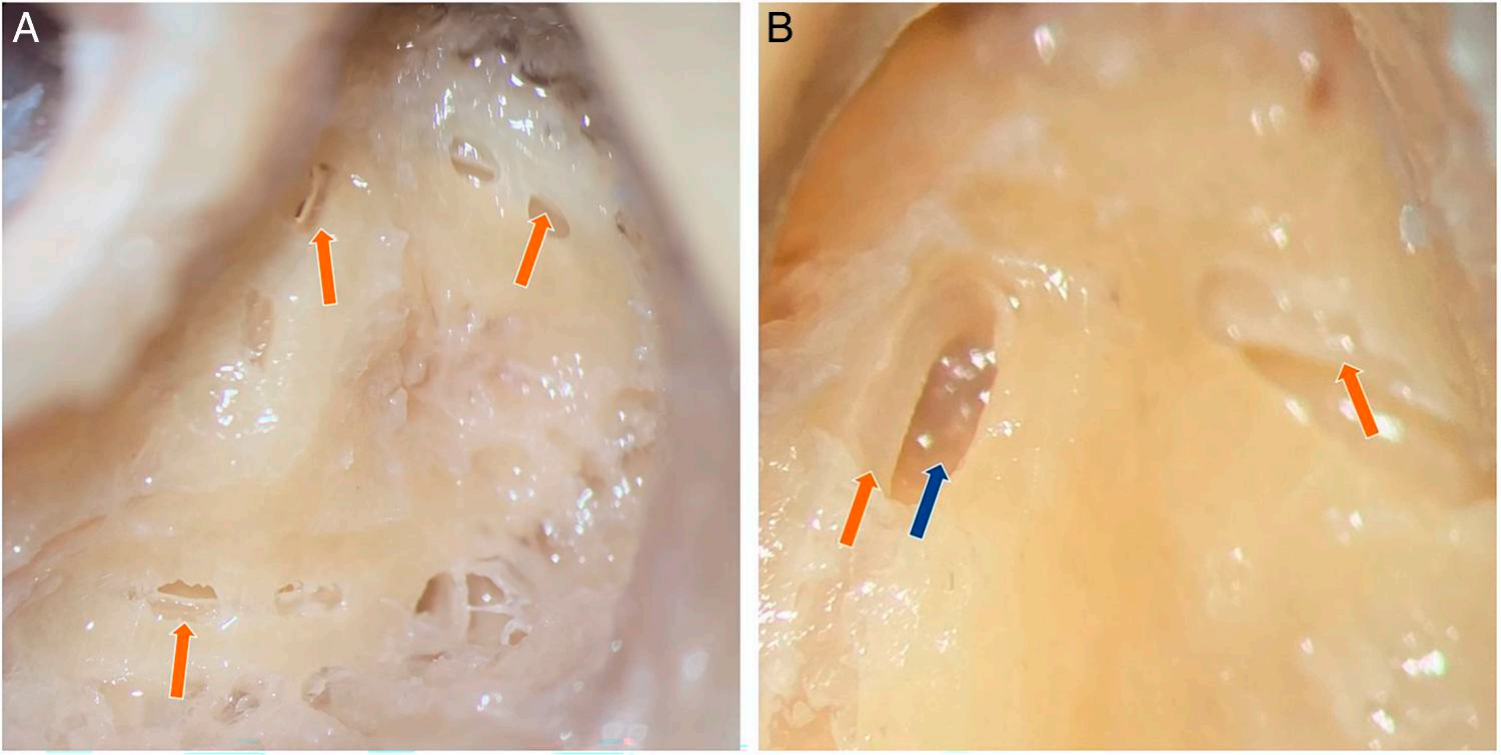
Microscopic view after piezoelectric drilling of 2 fenestrations in each semicircular canal. A, View of 3 semicircular canals. B, View after drilling extensively near the cupula. Orange arrows: membranous labyrinth. Blue arrow: cupula.

Ten temporal bones were included, evenly divided between left and right sides. Consequently, a total of 60 fenestrations were made in 30 semicircular canals. The fenestrations were drilled by 2 surgeons (J.S. and R.v.d.B., 6 and 4 temporal bones, respectively). In all 60 instances, the membranous labyrinth could be properly inspected through the fenestrations [(Fig. 1 and Video 1)]. In all instances the membranous labyrinth was intact (Table 1).

**Table 1:**
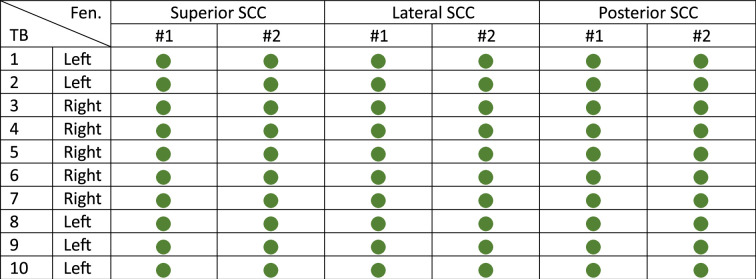
The Success of Membranous Labyrinth Preservation When Fenestrating the Semicircular Canals Using the Piezoelectric Drill

The results of the 10 temporal bones, with laterality and the success of preserving the membranous labyrinth. Each semicircular canal was fenestrated twice (results per fenestration in each column). Green dots represent successful fenestrations in which the membranous labyrinth remained intact. TB 1 to 5 and 9 were drilled by the first author (J.S.); 6 to 8 and 10 by the last author (R.v.d.B.). Fen. indicates fenestration; SCC, semicircular canal; TB, temporal bone.

## DISCUSSION

Piezoelectric drilling enabled consistent fenestration of semicircular canals in cadaveric temporal bones without damage to the membranous labyrinth in all fenestrations. This demonstrates that structural preservation of the delicate membranous labyrinth can be achieved reliably during bony fenestration. If these results translate to a clinical context, the approach could provide a foundation for function-preserving inner ear surgery. In vestibular implantation, this technique may open the door to treating patients with residual hearing.

A recent systematic review compared piezoelectric devices with rotating drills in ear and temporal bone surgery.^8^ It illustrates the variety of applications that have been investigated and highlights the benefits of piezoelectric bone removal near soft tissue. The ability to perform precise dissection at the bone-tissue interface while maintaining good visibility represents a clear advantage over rotating drills. Although a few studies reported on inner ear surgery, mainly regarding stapedotomy, no studies investigating semicircular canal surgery were identified.

In that review, 2 commercially available piezoelectric drill systems for otologic surgery were identified: Piezosurgery (Mectron S.p.A.) and Sonopet (Stryker Corporation, Kalamazoo, MI, USA), with some differences in design and functionality.^8^ The Piezosurgery system was selected for the present study, as the available inserts’ sizes and shapes were deemed most suitable for fenestrating semicircular canals with preservation of the membranous labyrinth.

Recently, a hand-guided robotic drill^9^ was investigated for the current purpose in a similar setting.^7^ It was set up to automatically stop drilling upon bony fenestration and showed feasibility in preserving the membranous labyrinth. Occasional inadequate drill bit fixation hampered consistent performance. Nonetheless, the study demonstrated that the drill’s principle functioned effectively. As it was designed to universally fit various drill bits in research settings, improved fixation can be incorporated in future designs. However, this drill is still in a preclinical phase, while piezoelectric surgery could be implemented rapidly, as piezoelectric instruments for otologic surgery have been commercially available for several decades.

Although piezoelectric surgery may preserve the membranous labyrinth, fenestrating the inner ear might still lead to hearing deterioration. Other factors than endolymph leakage might play a role, or piezoelectric drilling itself might induce it. Although the piezoelectric device operates at an ultrasonic frequency (ie, above the upper limit of human hearing), it generates noise at subharmonics of its working frequency (mainly 10 kHz).^10^ Yet, noise levels are similar to those generated by conventional rotating drills, and they are also influenced by the type of insert tip.^10^ To minimize noise-induced hearing loss, the optimal tip design should be chosen and the duration of exposure should be minimized.^10^ Nevertheless, when used on or near the otic capsule, piezoelectric surgery has yielded variable effects on sensorineural hearing.

Several studies on piezoelectric stapedotomy, all from the same group, reported no deterioration in sensorineural hearing (250 to 4000 Hz bone conduction thresholds and otoacoustic emissions up to 10 kHz).^11–13^ In contrast, 1 study reported a decline, with a mean deterioration of 11.5 dB at 4 kHz and 2/30 patients showing >15 dB deterioration in the pure-tone average (0.5 to 4 kHz).^14^ Notably, this latter group also reported deterioration of bone conduction thresholds >10 dB in 1 patient undergoing piezoelectric round window vibroplasty (15 and 25 dB at 2 and 4 kHz, respectively; audiometry 0.5 to 4 kHz presented).^15^ Following these findings, a study in rats observed damage to the organ of Corti at the lower basal region in all ears after drilling on the cochlear promontory.^16^ A study in chinchillas reported similar cochlear damage, along with deterioration in auditory brainstem thresholds and otoacoustic emissions, after drilling on an intact ossicular chain.^17^ These effects might be attributed to the aforementioned microvibrations, as well as to generated cavitation. Cavitation is the phenomenon in which rapid pressure changes create small vapor-filled cavities in the fluid. These bubbles oscillate, generating local pressure fluctuations and fluid flows that produce shear forces. These effects are highly localized and diminish rapidly with increasing distance from the cavitation site.^18^ This might partly explain why operating near the basal hair cells may contribute to damage and occasional high-frequency hearing loss. The impact on semicircular canal function remains largely unknown but is likely to be less pronounced, given the greater anatomical distance between their sensory epithelium and the investigated drilling sites. Conversely, fenestrations of the semicircular canals may also be sufficiently distant from the cochlea to minimize cochlear risk. In the specific context of vestibular implantation, hearing deterioration of up to 25 dB at a limited number of frequencies (as observed in Cuda and Colleagues^14,15^), could be considered relatively mild.^2^ Nonetheless, other factors may also contribute in this context, such as pressure changes during insertion and inflammatory processes. While electrode insertion into the semicircular canal is often assumed to inevitably rupture the membranous labyrinth, piezoelectric surgery has made it possible to observe that this is not necessarily the case. Video 2 shows the membranous labyrinth through a slit opening in the canal, with the electrode displacing it rather than rupturing it.

From these experiments, several practical considerations emerged regarding the effective use of the drill. First, to improve efficiency, the mastoidectomy and semicircular canal bluelining were performed with a regular rotating drill, with the piezoelectric drill used only briefly for fenestration. Second, the tip was moved along the canal rather than perpendicularly, creating a gradual, even thinning and a well-shaped fenestration. Although the curette-shaped insert was used, the direction of movement was mainly with the convex side facing the bone (like a ‘shoulder barge’), thereby sliding along the bony edges and avoiding hooking the membranous labyrinth (see Video 1). Third, during drilling, it was important to maintain contact with the bone and keep the tip moving to achieve smooth bone removal. Lastly, it was noted that some tiny pieces of endosteum or bone fragments remained attached to the membranous labyrinth, likely due to the connective tissue strands suspending the membranous labyrinth within the bony canals (see Video 3).

The results from this study were consistent across all semicircular canals, with 60 fenestrations performed in total. The position of the membranous labyrinth in relation to the fenestration—the membranous labyrinth is located along the outer curvature of the canal^19^ and therefore lateral canal fenestrations are made directly over the membranous labyrinth—did not appear to influence the outcome when using this drilling technique. Furthermore, the learning curve was short and the device appeared relatively intuitive to use. A brief test session was sufficient for surgeons to become familiar with the technique. Despite these strengths, certain limitations should be acknowledged. In this temporal bone study, specimens were preserved in formalin, which may alter the structure and strength of the membranous labyrinth, and the pressure within the post-mortem membranous labyrinth is likely reduced. Nevertheless, since formalin generally impairs tissue integrity,^20^ the membranous labyrinth would likely be more vulnerable to damage. Its integrity was assessed with the surgical microscope, and as such, cellular damage or microperforations cannot be ruled out. However, thanks to the absence of bone dust in piezoelectric surgery, the smooth and shiny surface of the tubular membranous labyrinth could easily be appreciated, consistent with its integrity. Nonetheless, the findings warrant validation in living subjects. In addition, a direct comparison with a conventional rotational drill (eg, using a diamond burr) was not included in the study design. This type of drilling is influenced by various surgeon-dependent factors, whereas piezoelectric drilling is based on the principle of tissue selectivity. This study therefore focused on assessing the ability of the piezoelectric drill to preserve the membranous labyrinth.

## CONCLUSION

Piezoelectric fenestration of the semicircular canals consistently preserved the membranous labyrinth in temporal bones. This is promising for semicircular canal surgery, particularly vestibular implantation and canal plugging. A next step would be to demonstrate its feasibility in living subjects, after which its impact on hearing and residual vestibular function could be studied.
